# Designing a physical activity intervention for children with asthma: a qualitative study of the views of healthcare professionals, parents and children with asthma

**DOI:** 10.1136/bmjopen-2016-014020

**Published:** 2017-03-24

**Authors:** Russell Jago, Aidan Searle, A John Henderson, Katrina M Turner

**Affiliations:** 1 Centre for Exercise, Nutrition and Health Sciences, School for Policy Studies, University of Bristol, Bristol, UK; 2 Biomedical Research Unit in Nutrition, National Institute for Health Research (NIHR), Diet and Lifestyle at the University Hospitals Bristol NHS Foundation Trust and the University of Bristol, Bristol, UK; 3 School of Social and Community Medicine, University of Bristol, Bristol, UK; 4 The National Institute for Health Research Collaboration for Leadership in Applied Health Research and Care West (NIHR CLAHRC West) at University Hospitals Bristol NHS Foundation Trust, UK

**Keywords:** physical activity, children, QUALITATIVE RESEARCH

## Abstract

**Objectives:**

Qualitative methods were used to examine: (1) the attitudes of health professionals to promoting physical activity for children with asthma; (2) reasons why children with asthma are less active and (3) how a physical activity programme for children with asthma could be designed.

**Design:**

Semistructured interviews were conducted with health professionals, children with asthma and their parents between October 2015 and March 2016. Interviews were transcribed verbatim and thematically analysed.

**Setting:**

Primary and secondary care in Bristol (UK).

**Participants:**

Interviews were held with 8 primary care practitioners (5 general practitioners, 2 nurse practitioners and 1 practice nurse), 9 parent–child dyads (2 fathers, 7 mothers, 6 sons, 3 daughters) of children aged 6–7 who had asthma and 4 secondary care professionals (2 respiratory consultants, 2 specialist nurses).

**Results:**

Health professionals reported that physical activity was beneficial for children with asthma and if managed appropriately, children with asthma could be as active as children without asthma. Current promotion of physical activity for children with asthma was limited and restricted by NHS staff time, access to inhalers at school and a lack of parental knowledge. Potentially important components of a new programme include parental education on the possibilities of activity for children with asthma and the difference between exercise-induced breathlessness and asthma symptoms. Other important elements include how to use inhalers as a preventive measure, coping with exacerbations and practical solutions (such as clearing sputum), managing transitions from warm to cold climates and general symptom control.

**Conclusions:**

There is a need to build on current asthma programmes to increase the support for children with asthma to be physically active. Future programmes could consider working more closely with schools, increasing parental knowledge and providing children with practical support to help be physically active.

**Strengths and limitations of this study**

- Data from primary and secondary care health professionals, as well as children with asthma and their parents.
- Triangulation of findings from across participant groups.
- Small sample size that may limit generalisability to other settings.
- Lack of information about the severity of asthma or physical activity levels of children interviewed.

## Introduction

Physical activity is associated with lower body mass, blood pressure, lipids and improved psychological well-being among children.^1–5^ A number of studies have shown that large proportions of children do not meet the UK's Department of Health's recommendation of an hour of moderate to vigorous physical activity per day. The early school years (ages 6–8 years) are a key period when children's physical activity behaviours are established.^3^ Increasing young children's (6–8 years of age) physical activity is therefore important for current and future health.

Over 1 million children in the UK are currently receiving treatment for asthma.^6^ ^7^ Systematic reviews have suggested that children with asthma engage in less physical activity than children who do not have asthma, and these differences may be more marked for girls than boys.^8–10^ In addition, a recent systematic review of epidemiological studies has shown that children with low levels of physical activity are at up to 35% increased risk of new onset asthma and/or wheezing.^11^ The same review also reported that more than 50% of the cross-sectional studies reported positive associations between low physical activity and childhood asthma.^11^

There is some evidence that physical activity programmes improve peak flow, reduce exercise-induced bronchoconstriction (exercise-induced asthma) and improve physical fitness and quality of life among children with asthma.^9^ ^12^ For example, there are studies that have shown that health promotion programmes can reduce asthma exacerbations through encouraging physical activity,^13^ pilot study level evidence of the potential utility of active play as a physical activity intervention for children^14^ and current work evaluating intense physiotherapy interventions.^15^ There are, however, no physical activity behaviour change programmes to increase physical activity in children with asthma.

Current guidance from the National Institute for Health and Care Excellence (NICE) recommends that children with asthma follow the general public health guidance of engaging in an hour of moderate to vigorous intensity physical activity per day but focus on managing their asthma while being active.^16^ There is a need for UK specific programmes to help children with asthma to be more physically active.

The UK Medical Research Council's (MRC) guidance on the development of complex interventions^17^ states that effectiveness is likely to be enhanced if intervention content is developed in a stepwise process. An important initial phase in this process is understanding the problem to be addressed and seeking input from key stakeholders to coproduce behaviour change programmes. The aim of this study was to explore: (1) health professionals' attitudes in relation to promoting physical activity in children with asthma; (2) reasons why children with asthma are less active and (3) how a physical activity programme could be developed.

## Methods

### Recruitment

We aimed to conduct indepth interviews with (1) primary care health professionals who treated children with asthma (ie, general practitioners and asthma nurses); (2) secondary care health professionals who treated children with asthma; (3) children with asthma who were between 6 and 8 years of age and (4) the parents of the children interviewed.

Seven primary care practices which varied in the sociodemographic characteristics of their patient populations were purposefully sampled across the city of Bristol, which is located in South-West England. The sampled practices were approached about the study through the local clinical research network. Four practices who agreed to support the study conducted database searches to identify 6–8 year olds diagnosed as having asthma, and posted study invitation letters and information sheets to parents of children identified. Reply slips and prepaid envelopes were enclosed with these documents. A total of 79 letters and information sheets were sent, with reminder letters sent 3 weeks after the initial letter. In total, 10 families returned reply slips. These four study practices were used to identify primary care practitioners for interview who were invited via letter to take part in an interview once the practice had agreed to join study.

Seven secondary care practitioners who treated children with asthma in the local Children's Hospital were identified by a senior professional in the healthcare trust and then approached through an email by AS to ask for their participation in the study.

All healthcare professionals and parents provided written informed consent to take part in the study. Parents provided written informed consent for their child's participation and the child provided verbal assent.^18^

### Data collection

Families returning reply slips were telephoned by AS to arrange an interview, which was conducted in the family home. Health practitioners were interviewed in person on university premises, or in surgeries. Topic guides were used to ensure consistency across the interviews and were designed in parallel to ensure key areas were explored with all three groups, which would later enable triangulation of study findings.

The health practitioner guide focused on: (1) how children with asthma are managed, including the information they already receive about being physically active; (2) how a physical activity intervention could be tailored to meet the needs of this target group and (3) assess any potential barriers that would need to be addressed to facilitate delivery of a future intervention. The parent and child guides focused on: (1) the information that the family have received in relation to physical activity; (2) identifying any concerns about physical activity and asthma; and (3) identifying key issues to consider in the design of a future physical activity intervention for children with asthma.

All interviews were conducted by AS, who is an experienced qualitative researcher with a background and PhD in health psychology. Interviews were audio-recorded, transcribed verbatim and checked for accuracy.

### Analysis

A sample of transcripts (ie, 1 consultant, 1 nurse specialist, 1 GP, 3 parents and 3 children) were read and reread by AS and KMT, who then met to discuss their overall impressions of the data and agreed that the data should be analysed thematically to allow comparisons to be made within and across the three data sets.^19^ AS and KMT then independently coded the sampled transcripts and met again to discuss their coding and to agree a coding frame for each interview set. Transcripts were then uploaded into NVivo (V.10, QSR, Southport, UK) and coded according to the relevant coding frame. Initially, each data set, that is, interviews with primary care practitioners, secondary care practitioners, parents and children, were analysed separately before comparisons were made between the accounts of each group. Illustrative quotes are presented to highlight themes in the interviews. The quotes have been tagged with details of the respondent, including the job of the health professional and their gender, and if a parent, the gender of the child that is being discussed in the interview.

## Results

### Participants

Semistructured, face to face, interviews were held with five general practitioners (1 male, 4 female), two nurse practitioners (female) and one practice nurse (female) from the four practices in primary care. In terms of secondary healthcare, interviews were held with two respiratory consultants (1 male, 1 female) and two specialist nurses (2 female). Nine parent–child dyads (2 fathers, 7 mothers, 6 sons, 3 daughters, range 6–7 years of age) were interviewed with the parents present during the child interviews. The duration of the interviews ranged from 6.37 to 18.25 (mean 11.06) min for children, 14.36 to 32.40 (mean 21.08) min for parents, 21.07 to 58.53 (mean 26.5) min for primary care health professionals and 19.10 to 56.20 (mean 33.4) min for secondary care health professionals. All interviews were held between October 2015 and March 2016. While the relatively small numbers of participants in each group meant we may not have reached data saturation in each group, it was felt that within the time frame available for the study, a sufficient number of interviews had been held to address the study's main aims and there was a convergence of information across respondent groups.

### Key themes

Four broad themes emerged from analysis of the data. (1) The role of primary care and schools in promoting physical activity for children with asthma. (2) Health professional and children's views about physical activity for children with asthma. (3) The reasons why children with asthma are less active and potential solutions. (4) The potential content of a physical activity programme for children with asthma.

### Theme 1: the role of primary care and schools in promoting physical activity

Health professionals explained that the time pressures NHS practitioners were under meant promoting physical activity for children with asthma was challenging. The primary care practitioners also stated that primary care tended to focus on the symptom control and medicinal aspects of the condition, and less on the management of asthma through using, for example, physical activity. Although the interview guide did not mention schools, the majority of the primary and secondary care interviews highlighted the key role of schools in promoting physical activity in children with asthma.

I mean, it's like long-term gain, isn't it? You're changing someone's lifestyle, and hopefully, having a long-term, lifelong outcome. Whereas the NHS, I feel, at the moment, is a bit like crisis management, because they just deal with what's going wrong now. (HP003, Female Nurse Specialist, Secondary care)

I think our concerns are probably more medical. I think we do have a tendency to focus on the medical aspects of it, so the medication, the asthma reviews, the annual reviews (HP010, Female GP, Partner)

What we're supposed to focus on—inhaler technique and whether they-re using inhalers (HP006, Male GP, partner)

I suppose if you could deliver it [physical activity intervention] at schools, possibly. (HP007, GP Female, Respiratory Lead)

Yeah, maybe we need more education and closer links with schools. I mean, I know that's expensive, but I think what parents tell school and what we would say to a school are often quite different things. …And if there was some kind of mechanism where we could feed into the school. (HP003, Secondary care, Female, Nurse Practitioner)

### Theme 2: health professional and children's views about physical activity for children with asthma

There was a strong feeling that, with a few exceptions such as those with severe asthma or severe conditions, there was no reason why a child with asthma could not be as active a child without asthma, if their condition was managed appropriately.

I mean, we do, always, when we're assessing someone in outpatients, try and reiterate to them that they shouldn't be different to anyone else. I mean, there are a few that are very unwell, but generally, we should be able to control their asthma so that they actually can participate the same as anyone else can. (HP003, Female, Nurse Specialist)

All the health professionals interviewed felt that physical activity was good for children with asthma and supported the idea of a physical activity programme for children with asthma.

I think it [PA intervention] would be an important thing to deliver. (HP003, Female Nurse Specialist, Secondary care)

It's really about symptom control and making sure that the child is managing and whether that's having an inhaler at school and taking it before exercising. (HP004, Female, Nurse Practitioner)

They don't like PE because of their asthma. Actually, it shouldn't really, with the right treatment, it shouldn't have any impact on their life at all. (HP006, Male GP, Partner)

I think anything that can help get messages out to parents both about consistency and managing asthma, but also about the importance of exercise and healthy diet as well. Yes so I think anything that helps professionals to get out those consistent messages will be really useful, really. (HP011, Female, Clinical commissioner)

I'm sure, yes, some behavioural work would be good, definitely, to readdress those ideas that they're going to do themselves harm, or they could be suffering more as a result of doing exercise, when we know that the vast majority of them won't. (HP010, Female, GP)

Several of the children interviewed reported that they felt their asthma did limit their ability to be physically active. For example, when asked if asthma stopped them from doing anything, one boy aged 7 years (C003) commented ‘Sports’. Similarly, another boy commented that when playing football, he rarely ran and focused on passing the ball.

Like football, I barely ever get to run around with it. I just pass it quite a lot. (C002, Boy 7 years of age).

However, some of the children commented that their ability to be physically active could limited because they had forgotten to use their inhaler, suggesting that they felt increasing inhaler use would facilitate physical activity.

I could run around more, because normally I forget to do my brown puffer. Sometimes when I'm trying to breathe, my heart starts hurting because I didn't do it. (C0022, Boy 7 years of age)

### Theme 3: reasons why children with asthma are less active and potential solutions

Health professionals and parents mentioned a range of reasons for why children with asthma were less active than children without asthma. These reasons related to poor management, lack of parental knowledge, access to inhalers at school and overly protective parents.

#### Poor management and lack of knowledge

Poor management was identified by all health professionals as a key issue, with many stressing that physical activity was possible with appropriate, proactive use of inhalers. Many health professionals and parents felt that parents had a lack of knowledge about asthma and what happens during an asthma attack, particularly the differences between an asthma attack and breathlessness from being active.

I think there's myths about, If you've got asthma, you shouldn't do the exercise. I think, there's ignorance, because they don't know. (HP002, Nurse Specialist, Female, Secondary care)

So try and advise them to use inhalers before they start their exercise or something like that. (HP012, Female, Secondary care Consultant)

Yes, that you can get good symptom control, and why it's important in terms of future

lung health, and growth, and receiving your maximum lung capacity in childhood. (HP007, Female GP, Partner)

Is this breathlessness? Is this an asthma attack? and whatever. What should I do? (HP002, Nurse Specialist, Female, Secondary care)

Yes because I do think there are still lots of old wives tales still attached to asthma and—Yes, you want to somehow dispel some of those myths. (HP008, Female GP, Respiratory lead)

#### Access to inhalers in schools

Many parents and health professionals mentioned school policies, which stated inhalers should be kept in the school office rather than with the child, as a barrier to the child engaging in physical activity.

they are not allowed to carry their own inhalers with them all the time, so maybe educating the teachers to say Be aware of that. (HP008, Female GP, Respiratory lead)

He's not allowed to keep his inhaler on him, which I need to speak to the school

about, because they've said that's not allowed. He's got to go to the school office. (P003, Mother of son, 7 years)

Children also talked about the fact they had limited access to their inhaler at school and described going to the office to receive medication.

I'll go to the office, and they give me my inhaler… a couple of days ago I had to go the office because I really couldn't breathe. (C003, Boy, 7 years of age)

Accounts from some of the children suggested that they would be reluctant to ask for their inhaler in front of peers, perhaps because they were worried about standing out and feeling different. For example, when asked what he would do if he was out of breath and needed an inhaler at school, one boy aged 6 years (C006) said “I would just sit down.” When probed further, as to whether he would tell a teacher about needing an inhaler, the same boy said “I haven't done that (ask for inhaler) at school …. I would just sit down.” However, accounts from other children suggested they would be quite happy to ask for their inhaler.

I go up to the teacher and I put my hand up to say, even in assembly there's like 60 people in there or like 70 or 50 or like. … (C002, Boy, 7 years of age)

Analysis of the data did not suggest that there were gender or age differences between the children who reported being reluctant to ask for their inhaler and those who were happy to do so, although this might be due to the narrow age range of the children interviewed and the fact that only three of nine children were interviewed were girls.

#### Parental concerns

Health professionals suggested that some parents were very concerned about their child's asthma and that this hindered options for physical activity. Some parents did describe themselves as overprotective and commented that they treated their child with asthma differently to their child without asthma.

I don't think the children express concerns, I think it's parents that express concerns. (HP008, Female, GP Partner, Respiratory lead)

I just get the feeling that there's a lot of loss of confidence amongst parents in managing it. (HP006, Male GP, Partner)

We're over protective, he's our only child. So therefore we'll probably drag him off sooner than later. So it's where his limits are, vs what we feel his limits are. (P005, Father of son, 6 years)

Well, just probably, you know, she probably watches a bit more telly than [Name] and probably spends more time on her iPad than [Name]. And relaxing a little bit more, I would say, then [Name] does. (Mother of daughter, 6 years)

### Theme 4: key content of a new physical activity programme for children with asthma

In terms of considerations for a future programme, participants highlighted five potential strategies to help children with asthma be more physically active. Themes 4.1 (education) and 4.2 (stronger links with schools) link directly to the issues discussed above. The last three subthemes (4.3–4.5) related to the programme's content, delivery mode and who should deliver a new programme.

#### 4.1 Education

Education was highlighted by primary and secondary care health professionals as essential for the management of childhood asthma and was identified as a key component of any future intervention.

It is about education. It's about re-education as well because it may well be that they were told that at the time but they were just trying to get on top of whatever was going on then. It's just re-enforcing how you need to manage the medication and relate it to how the child is. (HP004, Female, Advanced Nurse Practitioner)

I think education about what asthma actually is so that people are aware of the condition. Educating them that exercise and things isn't detrimental, in fact it's beneficial for the children to participate, and what to do. (HP006, Male GP, Partner)

#### 4.2 Link with schools

The primary and secondary care professionals articulated a desire for stronger links between schools and healthcare staff who are helping children and their parents to manage asthma.

If there was some kind of mechanism where we could feed into the school and say, Actually, this child can do sport along with everyone else. If they can't, we would like you to tell us or give us some feedback, so we can manipulate their background medication. (HP003, Secondary care, Female, Nurse Practitioner)

I think for me, it's giving the parents the language, a little bit, to talk to the staff at school or when they're at home. And to say that they should take their blue inhalers with them, so they've got it there. (HP009, GP, Female, Asthma lead)

#### 4.3 Practical solutions

Primary and secondary care professionals identified a number of simple strategies that could be adopted by children, with the support of their parents, to help to manage their asthma. These strategies could be combined with the broader education components described above as part of a new physical activity programme.

So it'll be about symptom management, kind of techniques would you use. (HP002, Female, Nurse Specialist, Secondary care)

Coping with exacerbations, and clearing sputum, and all those kinds of things, isn't it. (HP007, Female GP, Partner)

If they are going out from a warm house, in the winter, to a cold environment or they are exercising going from a hot classroom to a cold football pitch, that often triggers it. It's just educating them about that, I think. It would be helpful. (HP008, Female GP, Respiratory lead)

Even putting something [i.e scarf] over your mouth, if you're walking to school and whatever (HP002, Nurse Specialist, Female, Secondary care)

So in fact, sometimes, like a bit of—some deep breathing exercises. Just actually-thinking about your breathing; breathing in, breathing out. So just-simple things like that. It's not high tech. (HP002, Nurse Specialist, Female, Secondary care)

Yes, that you can get good symptom control, and why it's important in terms of future lung health, and growth, and receiving your maximum lung capacity in childhood. (HP007, Female GP, Partner)

#### 4.4 Delivery mode

In terms of delivery mode, there was a clear sense from both the healthcare professionals in primary and secondary care as well as the parent interviews that while the opportunity to network with other parents of children with asthma would be very useful, group-based sessions would be logistically challenging to organise.

Some people are seeming to prefer online because some people don't like to mix with other people. … I've had a group of people that feel really supported when they're with other people and do it together… It might be a little bit of both maybe, a bit of online, a bit of face to face. A bit of both, yes. Okay, that would be a novel approach. (HP004, Female Nurse practitioner, Primary care)

Yes, that's the challenge, is finding a time that parents can access it, and whether they would see it as a priority to go to it, I think. (HP007, GP Female, Respiratory Lead)

The parent and primary care interviews suggested that it would be good if the content could have a degree of flexibility to meet the variety of needs of the families and give the families the skills and knowledge to find solutions that worked for them in their context.

You also have that chance of parents meeting other parents with children of a similar condition, that's where conversation often starts, isn't it? (P001, Father of son, 7 years)

It's all about trying to get the people involved in doing the course, to come up with the answers themselves isn't it? … I think it's trying to promote stuff, so perhaps they can see themselves, the difference it makes. (HP009, Female, Nurse Practitioner)

#### 4.5 Who should deliver a new programme

Health professionals suggested that a mix of parents of children with asthma, who have experience of the condition, health professionals and physical activity specialists would be a good mix of people to deliver new physical activity programme.

What you're looking for is giving an individual the knowledge to be a healthcare educator in this situation. So if you looked at, say, new onset diabetics in adults and type two diabetes, there's a lot of interventions where-part of the taking out of the GP's hand, but that the primary group provides a day or a half day for, You've got diabetes. Come and meet 20 other people who've got diabetes. See-a nurse, nurse educator, and a dietician educator and understand your-disease. (HP001, Male, Respiratory Consultant)

Maybe someone who comes in from the gym in his sports kit looks a bit cooler, you know? Maybe, that might be more effective. (HP003, Female Nurse Specialist, secondary care)

## Discussion

Findings presented here indicate that health professionals and parents want to support children with asthma's participation in physical activity and children themselves wish to engage in regular physical activity. The data showed that for many children with asthma, physical activity is a challenge and there is a need for more consistent approaches to help this group to be more physically active. There was a clear sense from all the health professionals interviewed that physical activity is desirable for children with asthma and with a very limited number of exceptions, there is no reason why this group of children cannot be active. Current NHS support is focused on symptom control with limited opportunities to help children with asthma to be more physically active. Working in partnership with schools to find ways to increase access to inhalers was viewed as one key way to facilitating physical activity.

A recent systematic review and meta-analysis has shown that physical activity may reduce the risk of developing asthma in prospective studies.^12^ The same study also reported that in cross-sectional studies, higher physical activity was associated with lower asthma prevalence.^12^ However, due to the cross-sectional nature of the studies, reverse causality could not be assessed.^12^ Moreover, a lack of information during key periods, such as childhood the link between physical activity and asthma, is not conclusive.^12^ However, it is important to note that in adults with asthma, higher levels of physical activity are associated with improved asthma control.^20^ Adults who meet physical activity guidelines are 2.5 times more likely to have good asthma control than individuals who do not.^20^ Thus, there is some evidence that physical activity may protect against the development of asthma and that physical activity can aide asthma management. In 2013, it was estimated that the annual UK cost of asthma was £2.5 billion, of which £900 million was spent on direct healthcare costs.^21^ Using some of these funds to promote increased physical activity and therefore improved asthma control and increased physical activity has the potential to reduce health costs.

Findings presented in this paper reinforce international information on the factors that limit the opportunities for children with asthma to be physically active. For example, qualitative research with children aged 8–10 years in New York showed that school management of inhalers and poor asthma control were key factors that affected physical activity participation.^22^ Similarly, Brazilian data have shown that 96% of mothers of children aged 9–19 years and young people with asthma reported that they felt that physical activity was important for their child but 37% of the sample restricted their child's physical activity opportunities.^23^ The same study also reported that 37% of mothers reported that exercise was dangerous for children with asthma, but there was no evidence of an association between the severity of the child's asthma and his or her physical activity. Qualitative data from Scotland have shown that many parents and school staff were very anxious about children with asthma being physically active, and that physical activity was perceived as a threat to asthma control.^24^ Thus, there is a clear need to communicate to parents and school staff that physical activity is possible for children with asthma and the challenge. The challenge is to build on current efforts to find more effective, easier to implement and sustain ways of helping this group to be more active more often.

### Implications for a new intervention

Collectively, the information presented above has highlighted a number of key issues that could be addressed in a future intervention to help children with asthma to engage in physical activity. A summary of the key messages, which can be organised into three areas of education, practical solutions and delivery, is presented in table 1. In terms of education, there was a clear sense among the parents and the health professionals of a need for more information about what asthma is and the difference between an asthma attack and exercise-induced breathlessness, as well as reassuring information about what is possible in terms of exercise. The parents were also seeking guidance on how to facilitate physical activity for their children. Family-based content that addresses these issues and addresses parental concerns about physical activity has been developed for children without asthma.^25–31^ There is a need to ensure that materials and support which cover these issues are provided for children with asthma and their families.

**Table 1:** Key issues to cover in a physical activity programme for children with asthma

Education	What is asthma?
	What is possible in terms of physical activity?
	Differences between asthma attack and breathlessness
	Facilitating physical activity
Practical solutions	Symptom control—managing exacerbations
	Use of inhalers including timing in relation to physical activity
	Breathing exercises
	Clearing sputum
	How to talk to schools about inhalers
	Managing indoor/outdoor transitions in cold weather
Delivery	Sharing experiences with other parents
	Support and encouragement
	Flexibility and tailoring information to family needs
	Possible mixture of online and group support
	Delivered in partnership with schools
	Delivered by health professional and activity professional

In terms of practical solutions, information on how to manage symptoms and when to use inhalers in relation to exercise is information that many families would find helpful. There is also a need for new information for schools on how to allow the children to use their inhalers in school and how the parents can initiate those conversations from a position of informed confidence. Families also need guidance on effective methods of clearing sputum and simple strategies for managing the transition from indoor to outdoor environments, such as using a scarf to cover the mouth.

Participants' views on the mode of delivery of a new intervention were mixed. Many parents expressed a desire to engage with other parents and wanted an opportunity to receive peer support from other families but also highlighted that they would meeting face to face logistically challenging. Schools were identified as a potential solution to this contradiction and it may be the case that a delivery mode that includes the development of networks at the school with more remote online support may provide a compromise that maximises the potential of group support but is achievable within the confines of busy modern lives. In terms of staff delivering the sessions, the parents and health professionals indicated that they would welcome the authority of an experienced and knowledgeable health professional, such as asthma specialist nurse, but would also like to see someone with a background in physical activity to guide these elements of the session. Finally, the interviewees highlighted that it was critical that the parents felt supported and encouraged during any sessions and the overall tone was helpful rather than critical with a focus on empowering parents to help their children to be active.

### Strengths and limitations

Conducting interviews with healthcare professionals, children with asthma and their parents meant this study was able to explore the views of the key stakeholders in the care of children with asthma. In addition, the open-ended nature of the interview questions meant participants were able to raise issues that were salient to them and not previously considered by the researchers. The sample, however, was relatively small and drawn from a single UK city. In addition, only four of the seven practices that we approached joined the study and these four practices were all based in lower income neighbourhoods. All of these factors will limit the generalisability of the findings. Similarly, as we do not have data on participants who did not join the study, we are unable to assess the extent to which the participants in our sample represented the broader group of families with children who have asthma. Thus, further work is need to establish the generalisability of our findings and to explore, for example, whether they are applicable to families residing in relatively affluent areas. Future studies may also want to collect objective data on the severity of the participants' asthma and their levels of physical activity. We did not do this and doing so would have facilitated a deeper understanding of the context in which the views of participants were expressed.

## Conclusions

There is a need to better support children with asthma to be physically active. Current healthcare provision for childhood asthma in the UK is under pressure and there is a need to build on current asthma programmes to increase the support for children with asthma to be physically active. Future programmes could consider working more closely with schools, increasing parental knowledge and providing children with practical support to help be physically active as part of efforts to extend current UK provision.
